# Acute cerebral microbleeds detected on high resolution head CT presenting with transient neurologic events

**DOI:** 10.1016/j.cccb.2024.100207

**Published:** 2024-01-17

**Authors:** Abdullah Tawakul, Arturo R Toro, José Rafael. Romero

**Affiliations:** a Department of Medicine, Faculty of Medicine, Umm Al-Qura University, Makkah, Saudi Arabia; b Chobanian Avedisian School of Medicine, Boston University School of Medicine, 715 Albany Street, 72 East Concord St. Boston, MA 02118-2526, United States; c NHLBI's Framingham Heart Study, Framingham, MA, United States; d Department of Neurology, Boston University School of Medicine, Boston, MA, 725 Albany St, Boston, MA 02118, United States

**Keywords:** Cerebral small vessel disease, Neurological markers, Cerebral microbleed, Anticoagulation

## Abstract

**Introduction:**

Cerebral microbleeds (CMBs) are deposits of hemosiderin-laden macrophages that can be visualized on T2-weighted MRI sequences as small, ovoid areas of signal void. These markers represent hemorrhagic cerebral small vessel disease and are usually subclinical and asymptomatic. In these cases, we present two patients who presented with symptomatic, acute CMBs.

**Case description:**

Case 1 involves a 70-year-old male with history of diabetes, hypertension, hyperlipidemia, and obstructive sleep apnea. Five days prior to presentation, this patient reported a transient period of left upper extremity weakness. CT was performed and demonstrated a lesion on CT imaging consistent with an acute CMB in the R centrum semiovale.

Case 2 describes an 82-year-old female with history of hypertension, remote large ischemic stroke, and post-stroke epilepsy. Patient described an episode of prolonged left sided shaking consistent with prior seizures despite her consistently taking anti-epileptic drugs. On CT, a small hyperdensity was seen in the R thalamus/internal capsule region consistent with acute CMB.

**Discussion:**

These two examples demonstrate acute CMBs causing patients to demonstrate symptoms mirroring those of a TIA and experience breakthrough seizures. A TIA would normally be an indication for antiplatelet therapy. Though prior reasoning warns against anticoagulation in patients with CMBs, recent works including the SPS3 (Shoamanesh et al., 2017) and WAKE-UP (Schlemm et al., 2022) trials both showed that the presence of CMB did not significantly affect outcomes after initiating antiplatelet therapy. One should adopt a more personalized approach when deciding the therapeutic intervention of choice in patients with prior CMB.

## Highlights

• Here, we demonstrate symptomatic acute cerebral microbleeds (CMB).
• Acute CMB may mimic a transient ischemic attack or cause breakthrough seizures.
• Acute CMB may be visualized on high resolution head CT.
• New trials suggest one may safely anticoagulate patients with CMB.

## Introduction

Cerebral microbleeds (CMBs) are increasingly recognized markers of cerebrovascular pathology, attributed to hypertensive vasculopathy or cerebral amyloid angiopathy (CAA) depending on their topographic location [1]. CMBs represent focal accumulations of hemosiderin-containing macrophages, most frequently adjacent to small blood vessels and in some cases with minute areas of tissue necrosis [2]. They are readily identified as small areas of MRI signal void in T2*weighted sequences, [1] and are not typically seen using unenhanced head CT. CMBs are considered to be clinically covert, serving more as a subclinical marker for the risk of stroke and dementia, but recent reports have noted they can present with acute symptoms [3]. Recognition of acute microbleeds as the cause of an acute cerebrovascular syndrome is important as it may result in different treatment, particularly with the use of antiplatelet or anticoagulant therapy. We present a series of cases illustrating this scenario, where a patient presented with a transient ischemic attack (TIA) mimic and where acute CMB was demonstrated on high resolution unenhanced head CT.

### Case 1

**Fig. 1:**
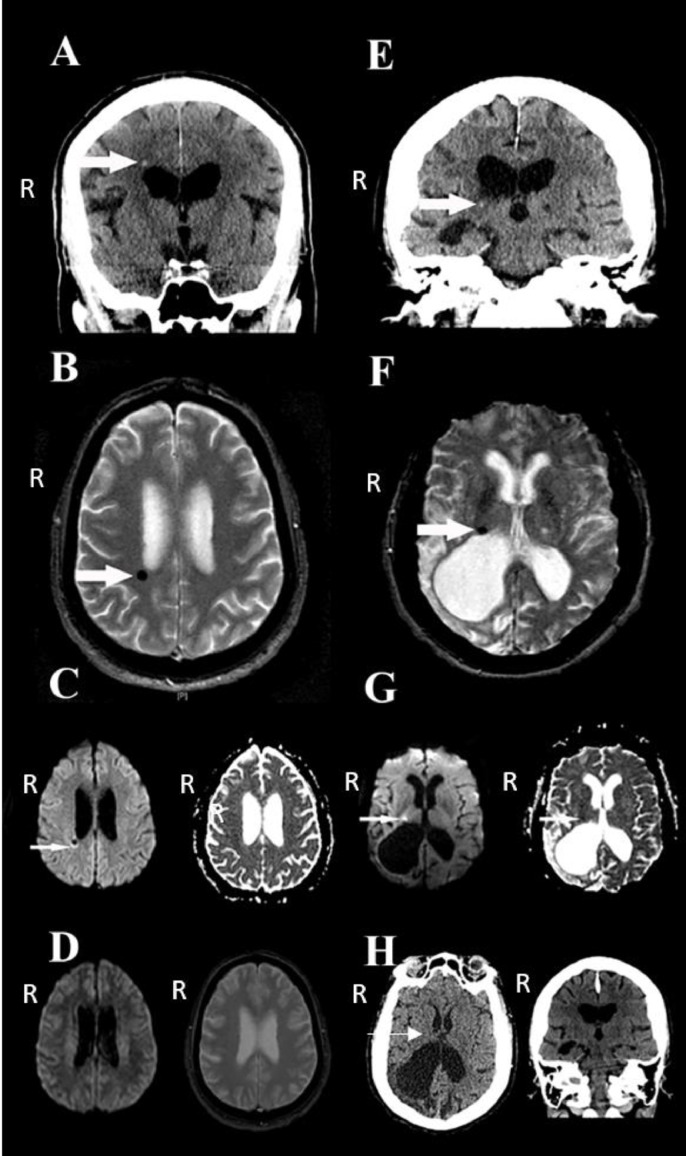
Case 1 images are in the left column (panels A–D) and case 2 in the right column (panels E–H). A and E. Coronal reconstructions using high resolution (1.25 mm slice thickness) unenhanced head CT demonstrates areas of small acute hemorrhage (arrow). B and F. Axial T2*GRE image demonstrating cerebral microbleeds (arrows). C and G. Axial diffusion weighted image (DWI) in the left and apparent diffusion coefficient (ADC) in the right for each case demonstrating hypointense lesion surrounded by hyperintense rim in case 1 (arrow) and hyperintense DWI signal with corresponding hypointense ADC signal for case 2 (arrows). D. DWI (left) and T2*GRE (right) images predating the occurrence of CMB show absence of the lesions for case 1. H. Axial and coronal high resolution (1.25 mm slice thickness) unenhanced head CT predating the occurrence of CMB in case 2 demonstrating absence of the acute hemorrhage (arrow).

A 70-year-old right-handed man with history of diabetes, hypertension, hyperlipidemia and obstructive sleep apnea presented with a transient episode of left upper limb weakness and numbness five days prior. The symptoms were present upon awakening from a nap and lasted for 20 min followed by complete resolution. He never had similar events and had no other neurologic symptoms. Neurologic exam in the emergency room was normal. A routine unenhanced head CT scan was unremarkable, however high-resolution head CT with coronal reconstructions (1.25 mm slice thickness) showed a small hyperdensity in the right centrum semiovale/corona radiata consistent with a small acute hemorrhage (Fig. 1A). Brain MRI confirmed on T2*GRE weighted images the presence of a CMB (Fig. 1B), which was not present on an MRI of the brain done 3 years earlier (Fig. 1D). The lesion was isointense on T1 weighted sequence (not shown) and demonstrated hypointense signal surrounded by a rim of hyperintense signal on the DWI sequence (Fig. 1C). All laboratory tests, including coagulation profiles, were normal. Upon review of all imaging studies, antiplatelet therapy was withheld in the treatment of this patient.

### Case 2

An 82-year-old right-handed woman with history of hypertension, prior remote large ischemic stroke with residual mild left hemiparesis and vision loss, and post-stroke epilepsy, presented with transient worsening of her pre-existent left sided weakness. The symptoms began suddenly while she was in the bathroom, with brief shaking of the left side followed by paralysis and fall to the ground. She had no loss of consciousness. The event was self-limited, with improvement to her baseline after arrival to the emergency room. The event was consistent with her prior partial motor seizures, but she was well controlled and compliant with antiepileptic treatments. Examination in the emergency room showed severe hypertension (blood pressure 200/130 mm Hg), mild left hemiparesis, vision loss consistent with her chronic deficits, and was otherwise unrevealing. A routine unenhanced head CT scan was unremarkable, however, high-resolution CT (1.25 mm slice thickness) showed a small hyperdensity in the right thalamus/internal capsule region (Fig. 1E), which was absent on a previous head CT scan from 2 years earlier (Fig. 1H). Brain MRI performed the same day showed a CMB in the same region as the lesion observed on the CT (Fig. 1F). The lesion demonstrated subtle restricted diffusion on DWI (Fig. 1G). Antiepileptic drug levels were in therapeutic range and all remaining laboratory tests were within normal limits. An EEG did not reveal epileptiform activity. The patient was treated to control hypertension, which was achieved gradually. Her chronic antiepileptic drugs and antiplatelet therapy were continued, the latter in view of her prior ischemic stroke.

## Discussion

The main findings in the present report are two-fold: first, they demonstrate that acutely developed CMB can present as a TIA mimic and be a cause of breakthrough partial seizures in a patient with controlled epilepsy, and second, they show that acute CMBs may be detected using high resolution unenhanced head CT.

The lesions fulfilled the criteria for diagnosis as CMB and were not suggestive of mimics [1]. The advantage in our cases is the availability of high-resolution CT, demonstrating findings consistent with acute hemorrhage; both cases had available prior imaging that did not reveal the acute lesions. In both cases, the lesion localization was consistent with the clinical presentation. In the first case the event presented as a transient ischemic attack and in the second case with a focal motor seizure. Both of our cases had history of hypertension and CMB were located in deep regions, suggestive of hypertensive vasculopathy as the underlying mechanism. In the second case there was a hypertensive crisis associated with the acute event and CMB, and responded well to blood pressure control.

CMBs are usually asymptomatic, detected incidentally on brain MRI, though they are not silent as they represent subclinical markers of heightened risk of stroke and dementia. Only recently have there been isolated case reports of CMB present as acute stroke or TIA symptoms [3,4]. Although transient neurologic symptoms attributed to CMBs have been recognized in patients with CAA without lobar intracerebral hemorrhage [5], we report transient neurological symptoms in patients with acute CMB due to hypertensive vasculopathy. Teo et al reported a case of CMBs presenting as TIA who subsequently developed a large intracerebral hemorrhage in the same CMBs location [3], but in their case high resolution head CT was not available. Our cases demonstrate the value of obtaining an MRI with GRE T2* weighted in acute neurological events such as TIA and unexplained breakthrough seizures in a patient with otherwise well controlled epilepsy since routine head CT scan may not be sensitive enough to detect small CMBs. We also report the utility of high-resolution unenhanced head CT in detecting these lesions, and confirming their acute nature.

The imaging characteristics noted on MRI are most suggestive of hemorrhage in acute stages. Although the changes noted on DWI sequences in the first case have not been previously reported on CMB, they are consistent with those seen in large intracerebral hematomas in acute to early subacute stages [6]. The hypointense center has been attributed to the magnetic field inhomogeneities caused by paramagnetic intracellular deoxyhemoglobin in acute hematomas, and intracellular methehmoglobin in early subacute hematomas, while the hyperintense rim may be due to T2-shine through effect by localized vasogenic edema [6]. In the second case, restricted diffusion was present at the site of CMB; this finding may reflect a hyperacute to acute stage of the hemorrhage as imaging was carried in the same day of presentation. A previous report by Atlas et al. showed similar findings in cases of large intracerebral hematomas, and were attributed to the MRI signal intensity patterns consistent with intracellular oxyhemoglobin, deoxyhemoglobin and/or methemoglobin [7]. Of note, a recent report also provided evidence of restricted diffusion in areas of microhemorrhage in patients with acute intracerebral hemorrhage [8]. Thus, the appearance of CMB on DWI sequences may vary as noted in the different patterns observed in the figure, which may also reflect differences in the hemorrhagic and ischemic (infarction) components of the lesions as has been suggested in pathological studies. Although the frequency of acute CMB mimicking an ischemic event syndrome is unknown, and probably low, this distinction may ultimately have therapeutic implications. Further studies will help clarify the clinical implications of CMB detection regarding decisions to start or continue antithrombotic or antiplatelet therapy, which could increase the risk of future devastating intracerebral hemorrhage.

Previously, hemorrhagic signs of cerebral small vessel disease have been considered a relative contraindication to using anticoagulation in treating embolic stroke of unknown origin (ESUS). Despite the absolute and relative risk of ICH increasing more rapidly with increased CMB burden, even with severe burden of CMB, the absolute risk of ischemic stroke is still greater [9]. The recent NAVIGATE ESUS trial found that the rate of ICH was not different between groups of participants with previous ESUS that were either prescribed aspirin or rivaroxaban [10]. The SPS3 trial, in contrast to previous studies indicating greater risk associated with anticoagulation treatment in the setting of pre-existing CMB, revealed no relationship between the presence of CMB and the outcome of dual or mono antiplatelet therapy in patients with previous stroke [11]. Similarly, the WAKE-UP trial showed that CMB presence did not cause a significantly increased risk of ICH and functional outcomes at 90 days were unchanged compared to participants without CMB [12]. These findings speak to the need for a comprehensive view when making considerations of the risks and benefits of therapies in patients with CMB. Patients with CMB are also at increased risk of ischemic events. Thus their detection should not be considered an absolute contraindication to antiplatelet or anticoagulant therapy when otherwise indicated.

## Funding sources

This work (design and conduct of the study, collection and management of the data) was supported by the Framingham Heart Study's 10.13039/100000050National Heart, Lung, and Blood Institute contract (N01-HC-25195; HHSN268201500001I) and by grants from the 10.13039/100000049National Institute on Aging (R01 AG059725, AG008122; K23AG038444; R03 AG048180-01A1; AG033193); 10.13039/100017751NIH grant (1RO1 HL64753; R01 HL076784; 1 R01 AG028321, P30 AG010129, NS017950), and 10.13039/100017540NHLBI grants (HL67288, and 2K24HL04334).

## CRediT authorship contribution statement

**Abdullah Tawakul:** Conceptualization, Data curation, Investigation, Methodology, Supervision, Writing – original draft, Writing – review & editing. **Arturo R Toro:** Investigation, Visualization, Writing – original draft, Writing – review & editing. **José Rafael. Romero:** Conceptualization, Data curation, Funding acquisition, Investigation, Methodology, Project administration, Supervision, Visualization, Writing – original draft, Writing – review & editing.

## Declaration of competing interest

The authors declare the following financial interests/personal relationships which may be considered as potential competing interests:

Jose Rafael Romero reports financial support was provided by NIH.
